# Chalinulasterol, a Chlorinated Steroid Disulfate from the Caribbean Sponge *Chalinula molitba*. Evaluation of Its Role as PXR Receptor Modulator

**DOI:** 10.3390/md10061383

**Published:** 2012-06-14

**Authors:** Roberta Teta, Gerardo Della Sala, Barbara Renga, Alfonso Mangoni, Stefano Fiorucci, Valeria Costantino

**Affiliations:** 1 The NeaNat Group, Department of Chemistry of Natural Products, University of Naples Federico II, Via D. Montesano 49, 80131 Napoli, Italy; Email: roberta.teta@unina.it (R.T.); gerardo.dellasala@unina.it (G.D.S.); alfonso.mangoni@unina.it (A.M.); 2 Department of Clinical and Experimental Medicine, University of Perugia, Via Gerardo Dottori 1, S. Andrea delle Fratte, 06132 Perugia, Italy; Email: barbara.renga@unipg.it (B.R.); fiorucci@unipg.it (S.F.)

**Keywords:** sterol sulfate, chlorine-containing steroid, *Chalinula molitba*, structure elucidation, PXR modulator

## Abstract

Chalinulasterol (**1**) a new chlorinated sterol disulfate was isolated from the Caribbean sponge *Chalinula molitba*. Its structure was elucidated using mass spectrometry and NMR experiments. The possible role of chalinulasterol as modulator of the PXR nuclear receptor was investigated but, in spite of the close structural relationship with the PXR agonist solomonsterol A (**2**), it showed no activity. The structural requirements for the PXR nuclear receptor activity were discussed.

## 1. Introduction

Sulfated sterols are a well-known class of secondary metabolites, often found in sponges and echinoderms [1,2], that are emerging as a new potential class of lead compounds in the research for new drugs. A recent paper [3] reports on the isolation from the sponge *Theonella swinhoei* of solomonsterols A (**2**) and B (**3**), tri-sulfated sterols having the cholestan skeleton. They differ from each other in the length of the side chain, and are among the few examples of truncated steroid derivatives isolated from marine sources. Both compounds have shown an important pharmacological activity, in that they are agonists of the PXR nuclear receptor [4]. PXR receptor is a transcription factor which is able to bind to a wide spectrum of structurally distinct endobiotic substrates and xenobiotic compounds, and is involved in innate immunity, xenobiotic metabolism, and detoxification [5,6]. PXR is proving to be an attractive target for small molecule drug discovery. In recent years, potential applications for exogenous PXR ligands have emerged in the treatment of important pathologies such as cancer [7] and inflammatory diseases [8].

**Figure 1:**
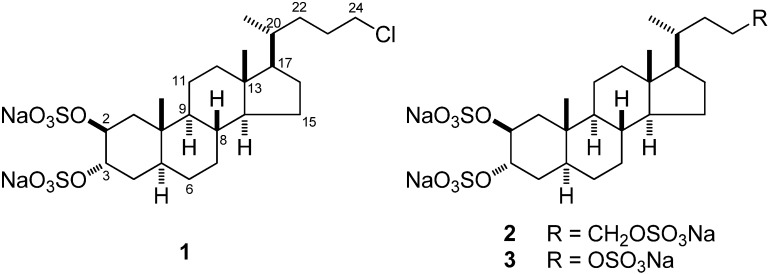
Structures of chalinulasterol (**1**) and of solomonsterol A (**2**) and B (**3**).

As part of our research program focused on the search for new anti-inflammatory and anti-cancer lead compounds from marine sponges [9,10], we analyzed the chemical composition of the Caribbean sponge *Chalinula molitba* (de Laubenfels, 1949), a light purple sponge that habits the mangroves of Little San Salvador (Bahamas Islands). This study led to isolation and structural identification of chalinulasterol (**1**) a new chlorinated sterol disulfate (Figure 1). Compound **1** has a close structural relationship with **2**, differing from the latter compound in having a chlorine atom instead of a sulfate function at position C-24 of the side chain. This relationship prompted us to investigate the possible role of **1** as modulator of the PXR receptors.

## 2. Results and Discussion

The positive-ion high-resolution ESI mass spectrum of **1** displayed a [M + Na]^+^ pseudomolecular ion peak at *m/z* 623.1447, in accordance with the formula C_24_H_39_ClS_2_O_8_Na_3_^+^ for this ion (calcd. 623.1462). The intensity of the (M + 2) isotopic peak in the MS spectrum (45%, calcd. 46.5%) and the peak at *m/z* 587.1675 ([M − HCl + Na]^+^) in the HRMS/MS spectrum confirmed the presence of a chlorine atom in the molecule. The MS/MS fragmentation pattern of **1** also revealed the presence of two sulfate groups from the peaks at *m/z* 503.1946 [M − NaHSO_4_ + Na]^+^, 262.8870 [2NaHSO4 + Na]^+^, and 244.8765 [Na_2_S_2_O_7_ + Na]^+^. The molecular formula was confirmed by the pseudomolecular ion peak at *m/z* 577.1669 observed in the negative-ion ESI mass spectrum, accounting for C_24_H_39_ClS_2_O_8_Na^−^ (calcd. 577.1678).

**Figure 2:**
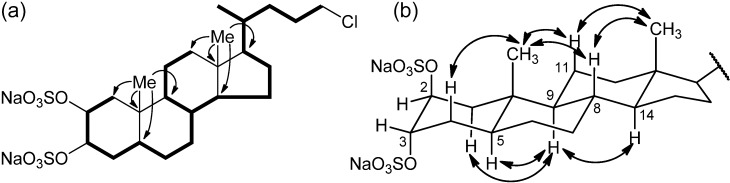
(**a**) Selected COSY and HMBC correlations of **1**, represented as bold bonds and arrows, respectively. (**b**) Selected ROESY correlations detected for **1**.

Inspection of the ^1^H NMR spectrum of compound **1** showed two methyl singlets at δ 0.69 (H_3_-18) and δ 1.01 (H_3_-19) and one methyl doublet at δ 0.95 (H_3_-21) suggesting its steroidal nature. The steroidal backbone could be assembled through the interpretation of COSY, TOCSY, HSQC and HMBC 2D NMR experiments (Figure 2 and Table 1). Analysis of the COSY and TOCSY spectra allowed the sequential assignment of all the protons of the tetracyclic system. The sulfate groups were located at position C-2 and C-3 because of the low-field resonances of H-2 and H-3 (δ 4.72 and 4.69) and of the respective carbon atoms C-2 and C-3 (δ 76.5 and 76.3). The HMBC correlation peaks of the methyl protons H_3_-19 with C-1, C-5, C-9, and C-10 and of H_3_-18 with C-12, C-13, C-14, and C-17 located the A/B and C/D ring junctions and completed the planar structure determination of the steroid ring system.

**Table 1:** ^1^H (700 MHz) and ^13^C (175 MHz) NMR data of chalinulasterol (**1**)in CD_3_OD.

Pos.		δ_H_ [mult. ^a^, *J* (Hz)]	δ_C_ [mult.]	Pos.		δ_H_ [mult. ^a^, *J* (Hz)]	δ_C_ [mult.]
1	α/ax	1.38 (dd, 14.6, 3.5)	39.1 (CH_2_)	13		-	43.8 (C)
	β/eq	2.12 (dd, 14.6, 2.1)		14		1.05 (m)	57.8 (CH)
2		4.72 (q, 2.7)	76.5 (CH)	15	α	1.59 (m)	25.2 (CH_2_)
3		4.69 (q, 2.7)	76.3 (CH)		β	1.08 (m)	
4	α/eq	1.66 (dt, 14.6, 2.7)	30.4 (CH_2_)	16	α	1.86 (ddd, 14.6, 9.4, 3.9)	29.3 (CH_2_)
	β/ax	1.79 (ddd, 14.6, 12.6, 2.7)			β	1.28 ^a^	
5		1.62 (tt, 12.6, 2.7)	40.3 (CH)	17		1.12 (q, 9.7)	57.5 (CH)
6	α/eq	1.25 (br. d, 14.5)	29.2 (CH_2_)	18		0.69 (s)	12.8 (CH_3_)
	β/ax	1.29 (qd, 12.7, 3.5)		19		1.01 (s)	14.2 (CH_3_)
7	α/ax	0.95 (qd, 12.6, 4.6)	33.3 (CH_2_)	20		1.44 (m)	36.6 (CH)
	β/eq	1.68 (dq, 13.0. 3.3)		21		0.95 (d, 6.5)	19.2 (CH_3_)
8		1.41 (qd, 11.2, 3.5)	36.5 (CH)	22	a	1.56 (dddd, 13.4, 10.6, 5.6, 2.9)	34.4 (CH_2_)
9		0.72 (ddd, 13.2, 10.5, 3.8)	56.7 (CH)		b	1.15 (dddd, 13.4, 10.6, 8.8, 4.3)	
10		-	36.4 (C)	23	a	1.81 (ddtd, 13.9, 11.2, 7.1, 4.5)	30.6 (CH_2_)
11	α/eq	1.53 (dq, 14.1, 3.5)	22.1 (CH_2_)		b	1.65 (ddq, 13.9, 10.9, 5.7)	
	β/ax	1.33 (qd, 13.2, 3.7)		24	a	3.53 (dt, 10.7, 6.5)	46.4 (CH_2_)
12	α/ax	1.14 (td, 12.6, 3.8)	41.4 (CH_2_)		b	3.51 (ddd, 10.7, 7.1, 6.4)	
	β/eq	1.99 (dt, 12.2, 3.5)					

^a^ Multiplicity and coupling constants of overlapping signals were determined using sections of the zTOCSY spectrum [11]; dt = doublet of triplets, td = triplet of doublets, dq = doublet of quartets, *etc.*

Information on the side chain was also provided by analysis of 2D NMR data. The COSY correlation between H-17 and the multiplet at δ 1.44 identified H-20; the latter was coupled with the methyl H_3_-21 and the protons at δ 1.15 and 1.56 (H-22a and H-22b), in turn coupled with the methylene protons at δ 1.65 and 1.81 (H-23a and H-23b). The coupling of H-23a and H-23b and the two protons at δ 3.51 and 3.53 (H-24a, and H-24b) could be also evidenced from the COSY spectrum. The linkage to this latter methylene group of the chlorine atom required from the molecular formula was shown by its ^1^H (δ 3.51 and 3.53) and ^13^C (δ 46.4) chemical shifts.

Analysis of the ROESY spectrum, supported by coupling constant analysis, defined the A/B, B/C and C/D *trans* ring junctions of a 5-α-cholane skeleton. The axial orientation of H-5, H-8, H-9, and H-14 was apparent from their respective coupling constants (Table 1), that of the angular methyl groups from the ROESY correlations of both H_3_-18 and H_3_-19 with H-8 and the axial H-11β. On this skeleton, the small coupling constants showed by H-2 and H-3 illustrated their equatorial orientation, and therefore the diaxial (*i.e.*, 2β,3α) orientation of the two sulfate groups. According to this information, the structure of chalinulasterol (**1**) was established as sodium 24-chloro-5α-cholane-2β,3α-diyl 2,3-disulfate.

**Figure 3:**
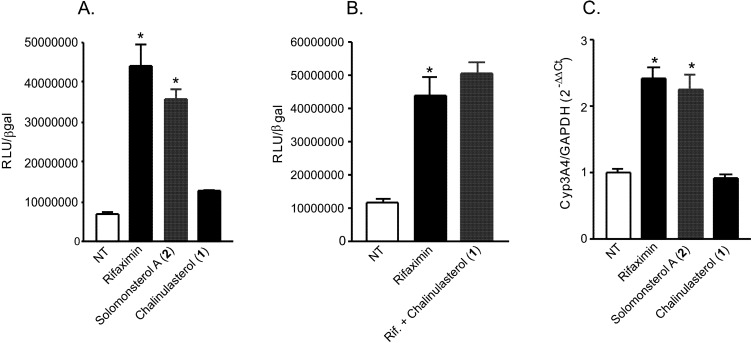
(**A**,**B**) Luciferase reporter assay. HepG2 cells, a hepatocarcinoma cell line, were transiently transfected with pSG5-PXR, pSG5-RXR, pCMV-βgalactosidase and p(CYP3A4)-TK-Luc vectors and then stimulated with (**A**) 10 µM rifaximin, solomonsterol A (**2**) or chalinulasterol (**1**) for 18 h, or (**B**) 10 µM rifaximin alone or in combination with 50 µM of compounds **1**. Relative Luciferase Units were normalized with β-galactosidase Units (RLU/βgal). NT, not treated. *P<0.05 *versus* NT cells. Data are mean ± SE of three experiments. (**C**) Real-Time PCR of Cyp3A4. HepG2 cells were stimulated with 10 µM rifaximin, **2** or **1 ** for 18 h. Total RNA was extracted and the relative mRNA levels of PXR target gene Cyp3A4, was measured. Values were normalized relative to GAPDH mRNA and are expressed relative to those of untreated mice, which are arbitrarily set to 1. Analysis was carried out in triplicate and the experiment was repeated twice. NT, not treated. *P<0.05 *versus* NT cells.

Considering the structural similarity with the PXR agonist solomonsterol A (**2**), we have investigated a possible role of chalinulasterol (**1**) in regulating the pregnane-X receptor activity and carried out a transactivation assay on HepG2 cells, a human hepatocarcinoma cell line as described in the Experimental Part. As shown in Figure 3A, despite the structural similarity with **2**, compound **1** failed in transactivating PXR. We have also investigated the possibility that **1** could act as potential PXR antagonist. As shown in Figure 3B, **1** failed to reverse the induction of luciferase caused by rifaximin, indicating that it was not a PXR antagonist. Similar results have been obtained by analyzing the effect exerted by **1** in terms of regulation of PXR mediated induction of Cyp3A4 gene. Indeed, **1** also failed to induce Cyp3A4. Although negative, these results have an important implication in terms of structure-activity relationship, because they suggest that the sulfate group present at position C-24 of compound **2** is essential in the ligand-receptor binding. This can be rationalized by the binding model of **2** to the PXR receptor proposed in [3], in which a clear interaction of the 24-sulfate with the positively charged Lys210 is observed, and further supports this model.

Although halogen-containing secondary metabolites are well-known and abundant in nature, particularly in marine organisms, only a few natural chlorinated steroids have been reported so far [12,13], and there is only one example in the literature of a sulfated and chlorinated steroid [14].

## 3. Experimental Section

### 3.1. General Experimental Procedures

High-resolution ESI-MS spectra were performed on a Thermo LTQ Orbitrap XL mass spectrometer. The spectra were recorded by infusion into the ESI source using MeOH as the solvent. Optical rotations were measured at 589 nm on a Jasco P-2000 polarimeter using a 10-cm microcell. CD spectra were recorded on a Jasco J-710 spectrophotometer using a 1-cm cell. NMR spectra were determined on Varian Unity Inova spectrometers at 700 and 500 MHz; chemical shifts were referenced to the residual solvent signal (CD_3_OD: δ_H_ 3.31, δ_C_ 49.00). For an accurate measurement of the coupling constants, the one-dimensional ^1^H NMR spectra were transformed at 64K points (digital resolution: 0.09 Hz). Through-space ^1^H connectivities were evidenced using a ROESY experiment with a mixing time of 450 ms. The HSQC spectra were optimized for ^1^*J*_CH_ = 142 Hz, and the HMBC experiments for ^2,3^*J*_CH_ = 8.3 Hz. High performance liquid chromatography (HPLC) separations were achieved on a Varian Prostar 210 apparatus equipped with a Varian 350 refractive index detector.

### 3.2. Collection, Extraction, and Isolation

Specimens of *Chalinula molitba* were collected in the mangroves of Little San Salvador (Bahamas Islands) during the 2010 Pawlik expedition. The samples were frozen immediately after collection and stored at −20 °C until extraction. The sponge (424 g of dry weight after extraction) was homogenized and extracted with MeOH (5 × 4 L) and then with CHCl_3_ (2 × 4 L). The MeOH extracts were partitioned between H_2_O and *n*-BuOH; the BuOH layer was combined with the CHCl_3_ extract and concentrated *in vacuo*.

The organic extract (10, 70 g) was chromatographed on a column packed with RP-18 silica gel. A fraction eluted with MeOH/H_2_O (8:2, 150 mg) was subjected to HPLC separation on a preparative RP-18 column [MeOH/H_2_O (6:4), Ascentis^®^ C18–25 cm × 10 mm, 5 µm-SUPELCO^®^], thus affording a fraction (1.4 mg) mainly composed of **1**.

Final purification was achieved by HPLC on an analytical RP-18 column (Ascentis^®^ C18—25 cm × 4.6 mm, 5 µm-SUPELCO^®^), using MeOH/H_2_O (6:4) as eluent, which gave 1 mg of pure compound **1**.

### 3.3. Chalinulasterol *(1)*

Colorless amorphous solid, [α]_D_^25^ +11.4 (*c* 0.1, MeOH); HRESIMS (positive ion mode, MeOH) *m*/*z* 623.1447 ([M + Na]^+^, calcd. for C_24_H_39_ClS_2_O_8_Na_3_^+^ 623.1462); MS isotope pattern: M (100%), M + 1 (27.5%, calcd. 27.8%), M + 2 (45.5%, calcd. 46.4%), M + 3 (11.5%, calcd. 12.2%,), M + 4 (4.9%, calcd. 5.4%); ^1^H and ^13^C NMR: Table 1.

### 3.4. Cell Culture

HepG2 cells were maintained at 37 °C in E-MEM containing 10% fetal bovine serum (FBS), 1% L-glutamine and 1% penicillin/streptomycin. HepG2 cells were stimulated 18 h with 10 μM rifaximin, **1** and compound **2**–**10** and relative mRNA levels of CYP3A4 were analyzed by Real-Time PCR. 

### 3.5. Transactivation Experiments

HepG2 cells were transfeted using Fugene HD transfection reagent (Roche). The plasmids used for luciferase assay were pSG5-PXR, pSG5-RXR, pCMV-βgalactosidase and the reporter vector p(CYP3A4)-TK-Luc. 48 h post-transfection cells were stimulated 18 h with 10 µM rifaximin, Solomonsterol A, compound **1** or with the combination of 10 µM rifaximin plus 50 µM compound **1**. Cells were lysed in 100 µL diluted reporter lysis buffer (Promega). 20 µL of cellular lysates were read using the Luciferase Substrate (Promega). Luminescence was measured using the Glomax 10/10 luminometer (Promega). Luciferase activities were normalized for transfection efficiencies by dividing the relative light units by β-galactosidase activity expressed from cotransfected pCMV-βgal.

### 3.6. Real-Time PCR

Total RNA was extracted using the TRIzol reagent (Invitrogen), purified of the genomic DNA by DNAase I treatment (Invitrogen) and random reverse-transcribed with Superscript II (Invitrogen). 50 ng template was amplified using the following reagents: 0.2 µM of each primer and 12.5 µL of 2× SYBR Green qPCR master mix (Invitrogen). All reactions were performed in triplicate and the thermal cycling conditions were: 2 min at 95 °C, followed by 40 cycles of 95 °C for 20 s, 55 °C for 20 s and 72 °C for 30 s in iCycler iQ instrument (Bio-Rad). The relative mRNA expression was calculated and expressed as 2^−(^^ΔΔCt)^. Primers used for qRT-PCR were: hGAPDH: gaaggtgaa ggtcggagt and catgggtggaatcatattggaa; hCYP3A4: CAAGACCCCTTTGTGG AAAA and CGAGGCGACTTTCTTTCATC.

### 3.7. Statistical Analysis

All values are expressed as means ± standard error (SE) of n observations/group. Comparisons of 2 groups were made with a one-wayANOVA with post hoc Tukey’s test. Differences were considered statistically signiﬁcant at values of *P* < 0.05.

## 4. Conclusions

The structure of chalinulasterol (**1**), a new chlorinated sterol disulfate isolated from the Caribbean sponge *Chalinula molitba*, was elucidated using mass spectrometry and NMR experiments. Chalinulasterol is only the second example of a sulfated and chlorinated natural steroid. The possible role of chalinulasterol as modulator of the PXR nuclear receptor was investigated, but, in spite of the close similarity with the strongly active solomonsterols **2** and **3**, resulted to be inactive. This suggests that the sulfate group present at the side chain of solomonosterols is essential in the ligand-receptor binding.

## Supplementary Files

Supplementary File 1: PDF-Document (PDF, 7564 KB)
