# Supplementary material for: Chalinulasterol, a Chlorinated Steroid Disulfate from the Caribbean Sponge Chalinula molitba. Evaluation of Its Role as PXR Receptor Modulator
Source: Mar Drugs. 2012 Jun 14;10(6):1383–90. doi: 10.3390/md10061383 (PMC3397446; doi:10.3390/md10061383)
Supplement: Supplementary File 1: — PDF-Document (PDF, 7564 KB) [file marinedrugs-10-01383-s001.pdf]

Electronic Supplementary Information

Table of contents

Figure S1. Negative-ion ESI mass spectrum of chalinulasterol (**1**) 2
Figure S2. Positive-ion ESI mass spectrum of chalinulasterol (**1**)................................2
Figure S3. Positive-ion ESI MS/MS spectrum of chalinulasterol (**1**) 3
Figure S4. ^{1}H-NMR spectrum of chalinulasterol (**1**)...3
Figure S5. COSY spectrum of chalinulasterol (**1**)..4
Figure S6. HSQC spectrum of chalinulasterol (**1**) – low-field region..........................5
Figure S7. HSQC spectrum of chalinulasterol (**1**) – high-field region.........................6
Figure S8. HMBC spectrum of chalinulasterol (**1**)...7
Figure S9. ROESY spectrum of chalinulasterol (**1**) 8

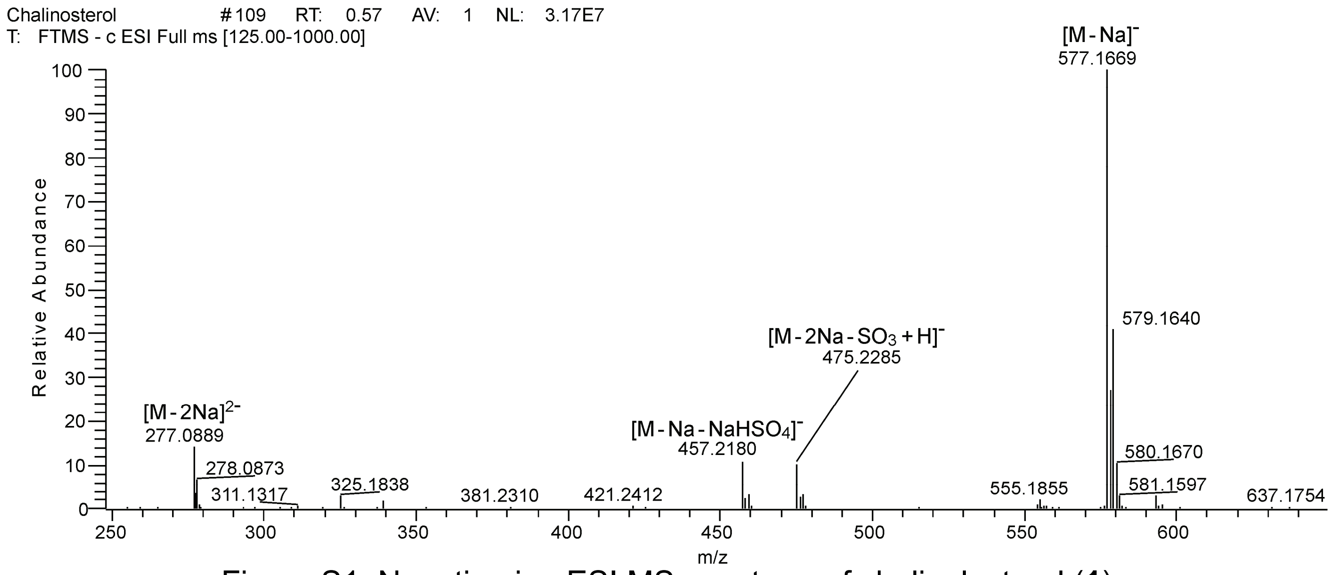

Figure S1. Negative-ion ESI MS spectrum of chalinulasterol (**1**)

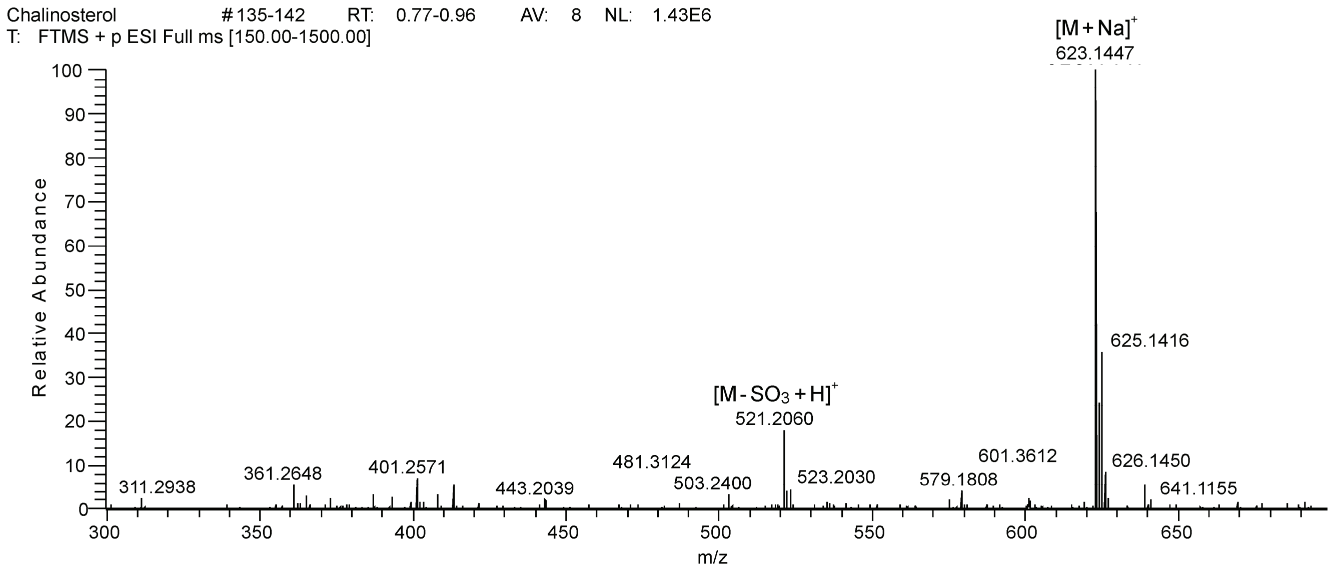

Figure S2. Positive-ion ESI MS spectrum of chalinulasterol (**1**)

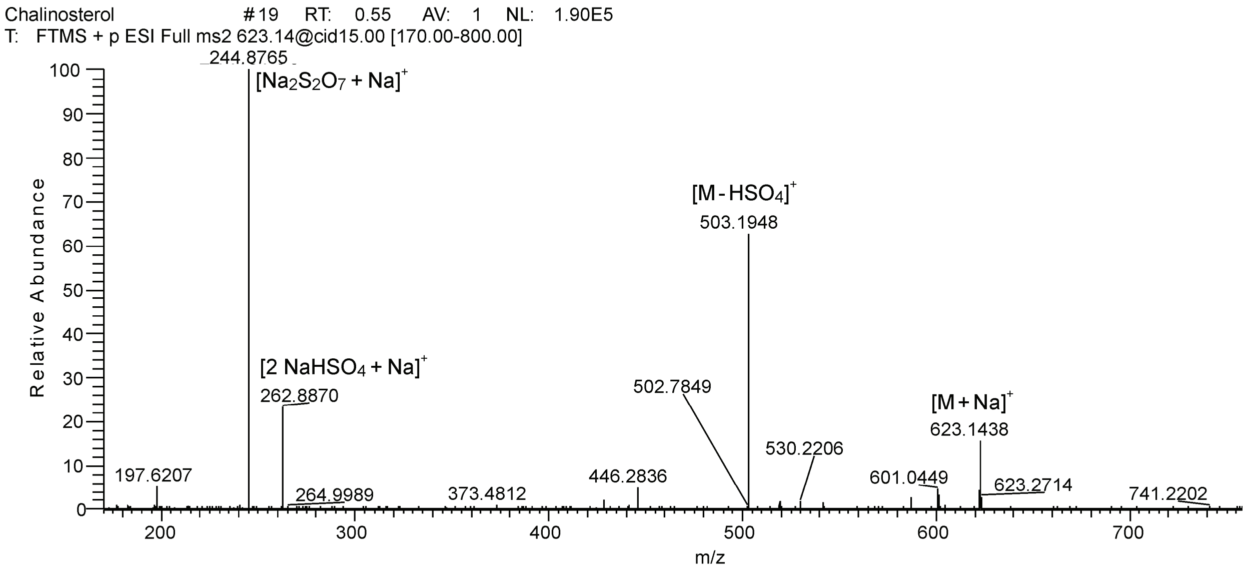

Figure S3. Positive-ion ESI MS/MS spectrum of chalinulasterol (**1**), parent ion at *m*/*z* 623

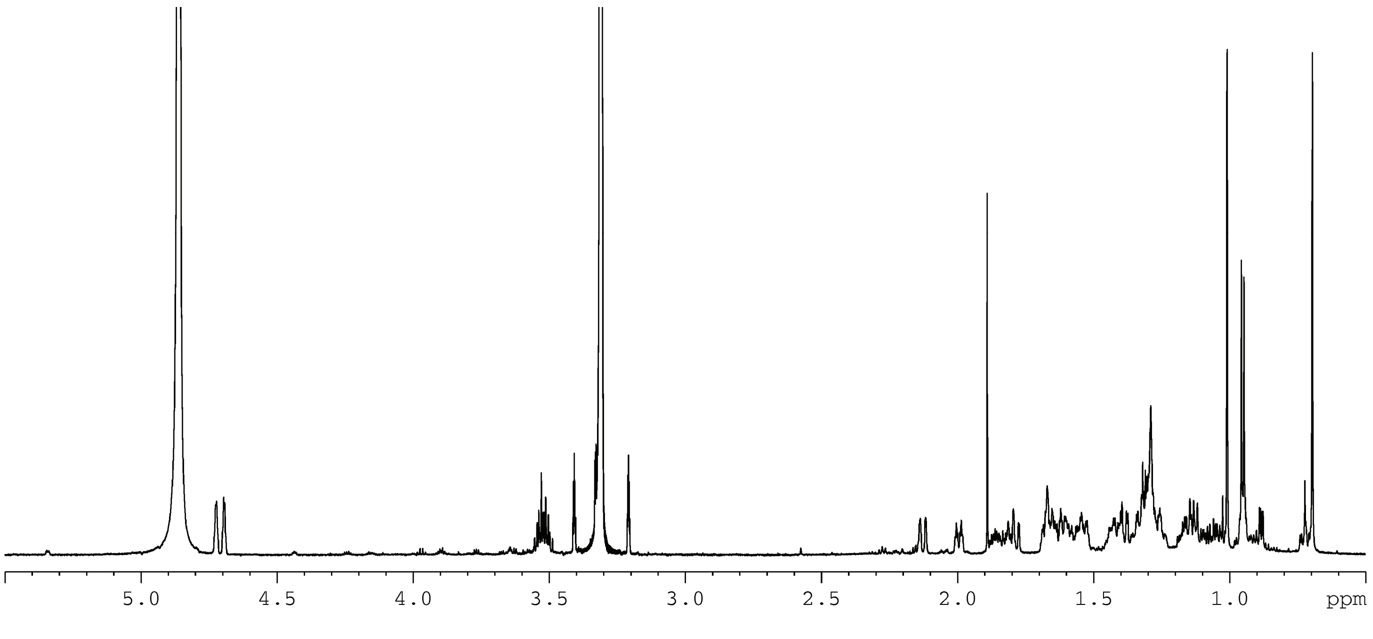

Figure S4. ^{1}H NMR spectrum of chalinulasterol (**1**) (CD_3OD, 700 MHz)

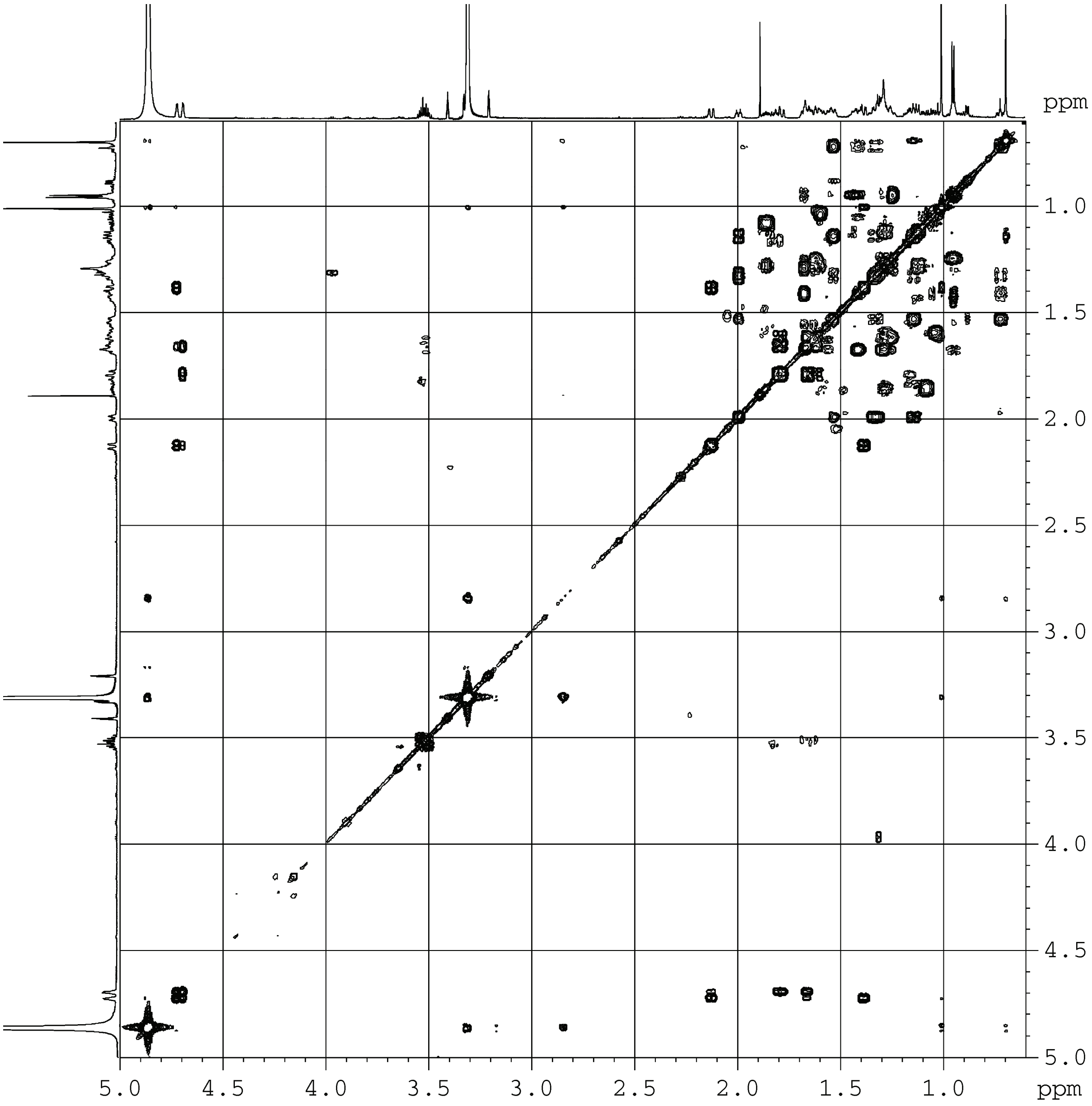

Figure S5. COSY spectrum of chalinulasterol (**1**) (CD_3OD, 700 MHz)

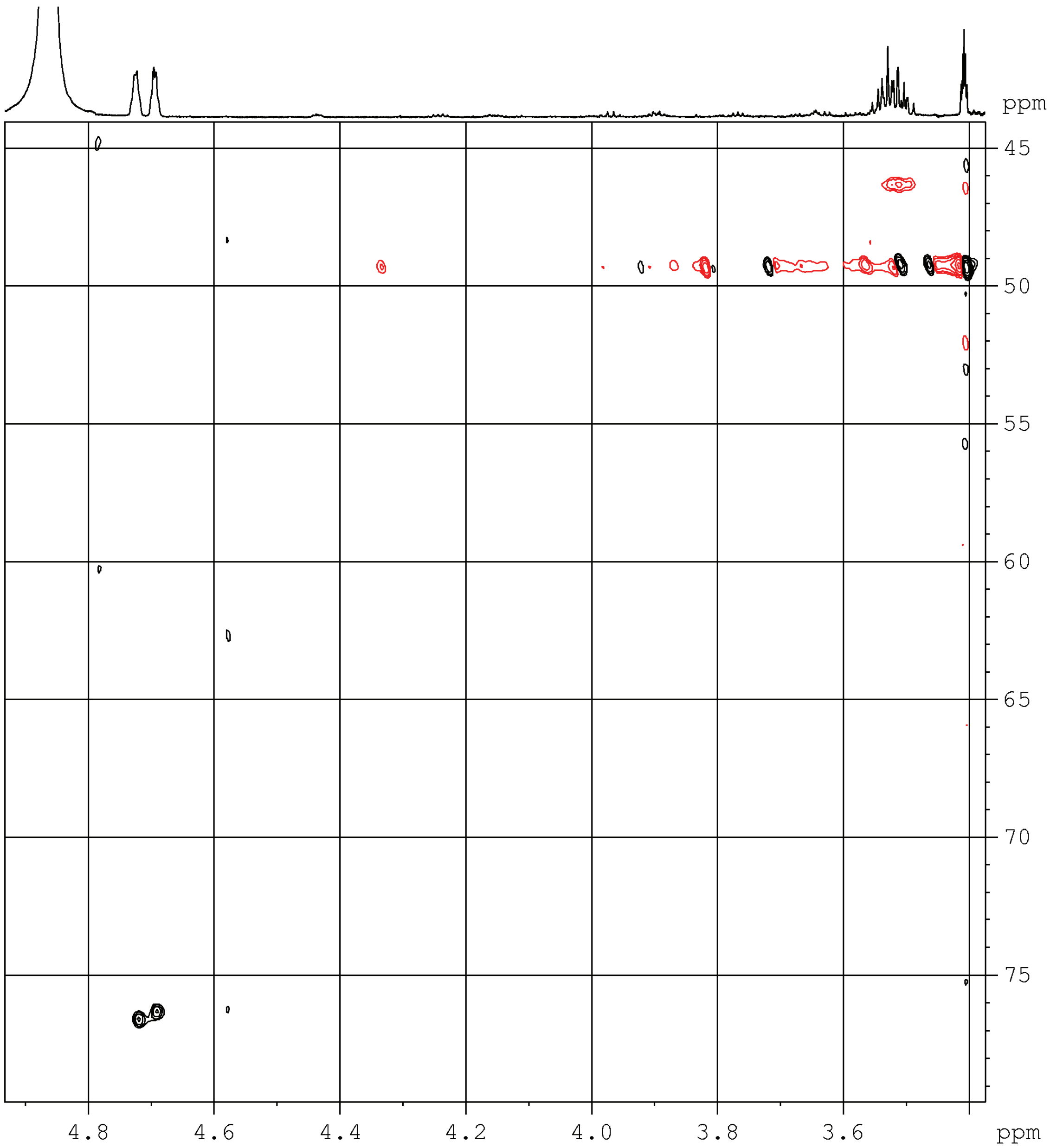

Figure S6. HSQC spectrum of chalinulasterol (**1**) (CD_3OD, 700 MHz) – low-field region

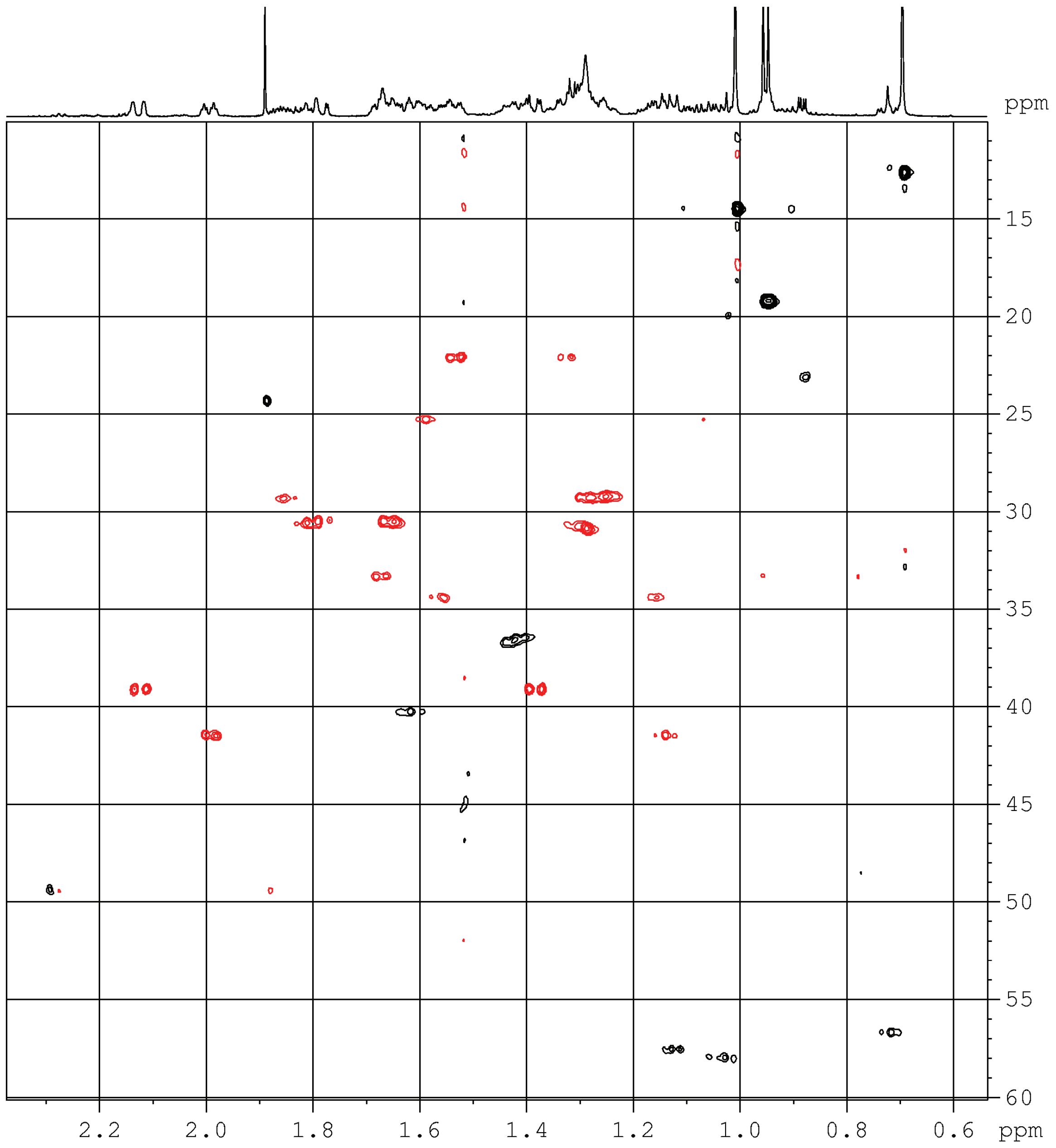

Figure S7. HSQC spectrum of chalinulasterol (**1**) (CD_3OD, 700 MHz) – high-field region

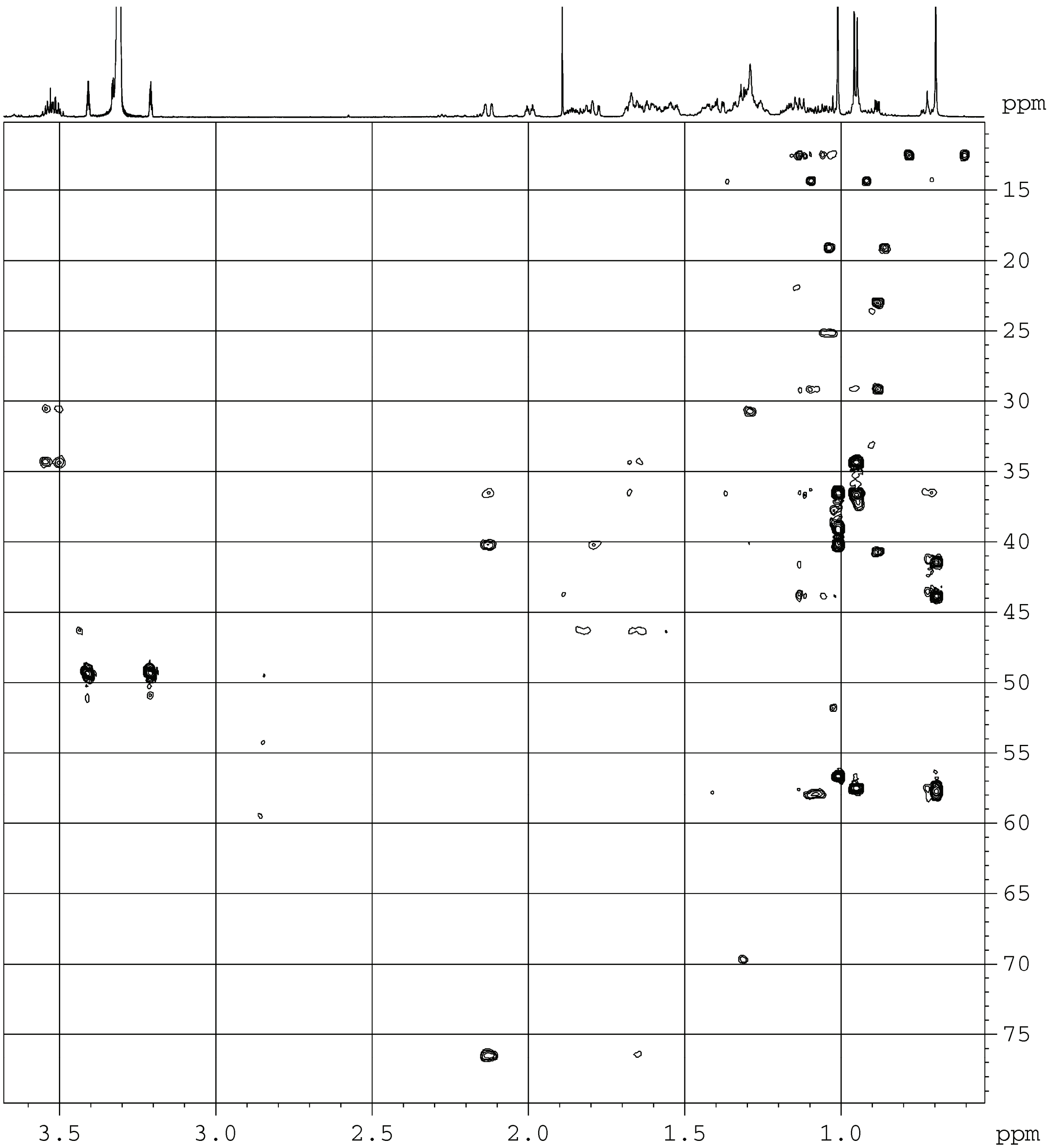

Figure S8. HMBC spectrum of chalinulasterol (**1**) (CD_3OD, 700 MHz)

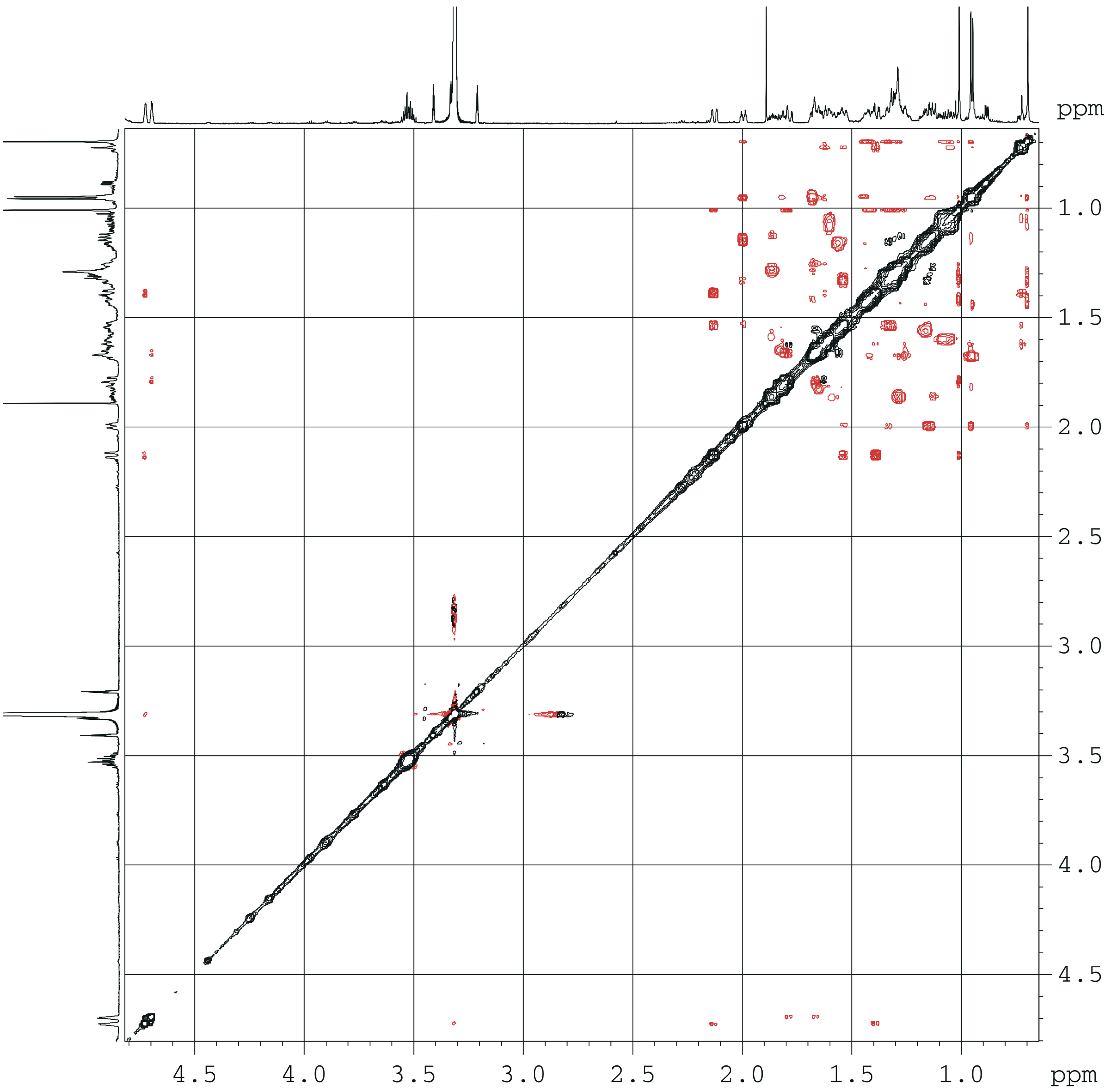

Figure S9. ROESY spectrum of chalinulasterol (**1**) (CD_3OD, 700 MHz)
